# Evaluation of Quantitative and Selective Sensory Fiber Dysfunction in Patients with Cirrhosis

**DOI:** 10.2174/0115672026289490240115075046

**Published:** 2024-01-26

**Authors:** Nan-nan Zhang, Zhi-yong Wang, Jian-min Chen, Zhi-peng Yan, Guo-xin Ni, Jun Ni

**Affiliations:** 1 Department of Rehabilitation Medicine, The First Affiliated Hospital, Fujian Medical University, Fuzhou 350005, China;; 2 Department of Rehabilitation Medicine, National Regional Medical Center, Binhai Campus of the First Affiliated Hospital, Fujian Medical University, Fuzhou 350212, China;; 3 Department of Rehabilitation Medicine, The First Affiliated Hospital of Xiamen University, Xiamen 361003, China

**Keywords:** Cirrhosis, current perception threshold testing, reference values, sensory nerve fibers, sensory peripheral neuropathy, primary biliary cirrhosis

## Abstract

**Background:**

Chronic liver disease has been reported to be associated with peripheral neuropathy. However, which sensory fibers are affected remains unknown. The objective of this study was to examine the function of sensory nerve fibers in patients with cirrhosis using the current perception threshold (CPT) test, as well as the correlation between blood biochemical indicators related to cirrhosis and CPT values.

**Methods:**

We recruited 44 patients with liver cirrhosis and 37 healthy controls of the same age and gender. The Neurometer^®^ system for the CPT test was used to stimulate the median nerve on the right index finger, as well as the deep and superficial peroneal nerves on the right hallux, using three distinct parameters (2000 Hz, 250 Hz, and 5 Hz). Comparative analysis was performed on the CPT values of the sensory nerves. Additionally, the correlation between CPT values and biochemical blood indicators in the study participants was analyzed.

**Results:**

Under 2000 Hz electrical stimulation, there was a significant difference between the cirrhosis and healthy control groups in the median nerve as well as the deep and superficial peroneal nerves (*p* < 0.05). In addition, the median nerve CPT value of the cirrhosis group was significantly higher than that of the control group at an electrical stimulation frequency of 250 Hz (*p* = 0.005). There was no correlation between CPT values and blood biochemical indicators.

**Conclusion:**

According to the results, the sensory peripheral neuropathy in liver cirrhosis is mainly manifested as Aβ fiber neuropathy.

## 1 INTRODUCTION

Cirrhosis has been reported to be accompanied by peripheral neuropathy (PN) [1]. Previous research indicated that the prevalence of PN in chronic liver disease ranges from 19% to 80% [2-4]. Several studies have indicated that small-fiber dysfunction (cooling threshold) is more prevalent than large-fiber dysfunction (vibration threshold) [2]. However, another study found that the rate of abnormal nerve conduction in large fibers was greater than the rate of abnormal thermal perception, indicating greater involvement of C fibers than large-fiber involvement [5]. However, it is still unknown as to which type of sensory fibers are affected by liver cirrhosis.

In this regard, it is essential to investigate quantitative approaches to the function of sensory nerve fibers in cirrhosis. In numerous fields, the current perception threshold (CPT), a type of quantitative sensory test, has been utilized to evaluate the function of sensory nerve fibers. Using different frequency alternating currents, it can be used to evaluate the three subtypes of nerve fibers (Aβ, Aδ, and C fibers) independently [6]. In particular, the 2000 Hz stimulus is used to assess the response of Aβ fibers, 250 Hz for Aδ fibers, and 5 Hz for C fibers [7]. In diabetes, it has been utilized as a complementary method for diagnosing and assessing diabetic PN symptoms [8]. The CPT test detects not only advanced severe neuropathy but also asymptomatic neuropathy in its earliest stages [8]. Patients in the early stages of Parkinson's disease, as determined by the CPT, can develop PN manifesting as Aδ and C nerve fiber neuropathy [9]. Recently, our research team has examined quantitative and fiber-selective evaluation for central post-stroke pain using CPT [10]. To our knowledge, however, there are few publications on its application in cirrhosis. Relatively few studies have investigated the association between CPT levels and biochemical blood indicators in patients with primary biliary cirrhosis (PBC). However, their findings have been inconsistent [3, 11].

The purpose of this study was to compare the sensory changes in patients with cirrhosis and healthy controls using the CPT test. In addition, the correlation between cirrhosis-related biochemical blood markers and CPT values was investigated.

## 2 MATERIALS AND METHODS

We utilized a cross-sectional design in this observational study. Between November, 2022 and March, 2023, we recruited 44 patients with liver cirrhosis and 37 age and gender-matched healthy controls from the Department of Hepatology at the First Affiliated Hospital of Fujian Medical University and the surrounding communities. All study participants were invited to undergo CPT testing at the hospital. Cirrhosis was diagnosed clinically with the assistance of supporting laboratory tests, ultrasonographic evidence of cirrhosis-shrunken or nodular liver, portal vein dilation, splenomegaly, ascites, or endoscopic evidence of esophageal varices. Blood biochemical indicators of patients with cirrhosis, including total bilirubin (TBil), albumin, aspartate aminotransferase (AST), alanine aminotransferase (ALT), alkaline phosphatase (ALP), and prothrombin time (PT), were routinely detected in the hospital using standard assays and methods. The Child-Turcotte-Pugh (CTP) score was used to determine the severity of cirrhosis, and patients were classified into three subgroups:

- Child-Pugh Class A Group: 14 patients with CTP scores of 5–6.
- Child-Pugh Class B Group: 14 patients with CTP scores of 7–9.
- Child-Pugh Class C Group: 14 patients with CTP scores of ≥ 10.

Inclusion criteria for patients with cirrhosis included age between 18 and 80, a clinical diagnosis of cirrhosis, and the capacity to understand testing instructions. The exclusion criteria included metabolic diseases like diabetes mellitus, central nervous system diseases like overt hepatic encephalopathy, stroke or Parkinson's disease, other causes of neuropathy like toxin or drug exposure, cervical or lumbar spondylosis, limb injuries, the presence of skin lesions at the testing site, renal impairment (elevated serum creatinine), and malignancy. None of the healthy controls had liver or other systemic diseases. Other exclusion criteria were identical to those applied to patients with cirrhosis.

This research was conducted in accordance with the Declaration of Helsinki and with approval from the Ethics Committee of the First Affiliated Hospital of Fujian Medical University (Approval No. MRCTA, ECFAH of FMU [20^20^]042). Prior to the study, informed consent was obtained from every participant.

### 2.1 Current Perception Threshold

We utilized the Neurometer^®^ CPT (Neurotron, Baltimore, MD, USA). The participants were evaluated while remaining seated in a quiet room with an ambient temperature of 24°C ± 1°C. For electrical stimulation, a pair of 1 cm-diameter, gold-plated, circular electrodes were applied, one on the lateral side and one on the medial side, of the right index finger distal interphalangeal joint and the right hallux interphalangeal joint [12]. We measured the median nerve (C7 dermatome) of the right index finger as well as the deep and superficial peroneal nerves (L4/5 dermatome) of the right hallux [13]. The threshold was determined by stimulating the participants with sinusoidal electrical currents of three different frequencies. For the purpose of measuring CPT, participants were required to identify the presence or absence of a stimulus using a forced choice protocol. After establishing a preliminary, tentative threshold, we administered a variety of stimuli near the provisional threshold to confirm the consistency and reproducibility of the threshold measurements. The CPT examination test was administered to all participants by the same operator.

### 2.2 Statistical Analysis

SPSS Statistics was utilized to conduct statistical analysis (version 22). The categorical variables were recorded as frequencies (percentages). The distribution was evaluated using the Kolmogorov-Smirnov test for normality with a single sample. Normally distributed variables are presented as mean ± SD and non-normally distributed variables as median (interquartile range). We used the t-test or Wilcoxon's signed-rank test to compare the CPT values of the cirrhosis and healthy control groups. Variance analysis or the Kruskal-Wallis H rank sum test was used to examine statistical differences in CPT values between patients with cirrhosis and healthy controls. Pearson’s or Spearman’s rank correlation analysis was used to test for a statistically significant relationship between the CPT values and blood biochemical indicators in the three subgroups. A P-value of < 0.05 was regarded as statistically significant.

## 3 RESULTS

### 3.1 Demographic and Clinical Characteristics

We enrolled 44 patients with cirrhosis (mean age: 52.52 ± 15.36 years; women, 11 (25%)) and 37 healthy controls (mean age: 46.86 ± 11.38 years; women, 12 (32.43%)). Age and gender were not significantly different between the cirrhosis and control groups (*p* > 0.05). The demographic and clinical characteristics of the participants are presented in Table **1**. In the majority (24 out of 44, 55.5%) of study participants, hepatitis B infection was the likely cause of cirrhosis, followed by alcohol in 13.6% (6 out of 44) of patients, both alcohol and hepatitis B infection in 11.4% (5 out of 44), and cryptogenic in 9.1% (4 out of 44). The clinical profile of the study participants revealed that splenomegaly was the most common physical finding, occurring in 65.9% of patients, followed by ascites, upper gastrointestinal bleeding, and pedal edema, which occurred in 59.1%, 34.1%, and 15.9% of patients, respectively.

### 3.2 CPT Parameters

#### 3.2.1 CPT Values in Patients with Cirrhosis

Sixteen patients (36.4%) had at least one abnormal sensory nerve threshold, with 9 patients (20.45%) having hyperesthetic type and 7 patients (15.91%) having hypoesthetic type sensory nerve dysfunction. Nine patients had sensory peripheral neuropathy (SPN) involvement in the upper extremities, and 9 had abnormal CPT values in the lower extremities. Child-Pugh Classes A, B, and C had SPN prevalence rates of 50%, 28.6%, and 31.3%, respectively. The incidence of SPN did not differ significantly between the three subgroups of the study (*X2* = 1.67, *p* = 0.433). As shown in Fig. (**1**), the cirrhosis groups had significantly higher CPT values at each frequency than healthy control groups. At 2000 Hz, statistically significant differences were observed between the median nerve and the deep and superficial peroneal nerves (*p* < 0.05). In the median nerve testing at 250 Hz, the CPT values of the cirrhosis groups were significantly higher than those of the healthy control groups (*p* = 0.005). There were no statistically significant differences between the two groups in the other frequency distributions.

#### 3.2.2 Comparison Among the Different Groups of Cirrhosis **and Healthy Controls**

In the deep and superficial peroneal nerves testing at 2000 Hz, the differences between the healthy controls and Child-Pugh class A group and the healthy controls and Child-Pugh class C group were statistically significant (*p* = 0.012, *p* = 0.045, Table **2**). There were no significant differences in CPT values between the median nerve and the deep and superficial peroneal nerves in the three study subgroups.

### 3.3 Relationship between Blood Biochemical Indicators and CPT Values

In the hepatitis B cirrhosis group (n = 24), there was no correlation between blood biochemical indicators and CPT values. In the alcoholic cirrhosis group (n = 6), CPT values in median nerve testing at 2000 Hz were positively correlated with ALT levels (r = 0.813, *p* = 0.049), whereas CPT values in median nerve testing at 5 Hz were negatively correlated with ALP levels (r = -0.828, *p* = 0.042). In the hepatitis B with alcoholic cirrhosis group (n = 5), CPT values in the median nerve testing at 5 Hz were positively correlated with TBiL levels (r = 0.984, *p* = 0.002), and CPT values in the deep and superficial peroneal nerves at 5 Hz were positively correlated with AST (r = 1.000, *p* < 0.01) and ALP levels (r = 1.000, *p* < 0.01). However, there was no correlation between the blood biochemical indicators and CPT values of the cirrhosis groups.

## 4 DISCUSSION

In this cross-sectional study, most patients with liver cirrhosis did not complain of paresthesia, dysesthesia, or weakness. However, the CPT values in the median nerve (C7 dermatome) and the deep and superficial peroneal nerves (L4/5 dermatome) tested at 2000 Hz were significantly higher than those in the healthy control groups, indicating that the CPT test can detect early asymptomatic neuropathy in patients with cirrhosis and that SPN primarily manifests as Aβ fibers neuropathy. It also indicates that the CPT values at 2000 Hz in these nerves have diagnostic value for distinguishing healthy individuals from patients with cirrhosis.

Aβ fibers transmit cutaneous touch, pressure, and vibration sensations [6]. Yoon *et al*. and Mapoure *et al*. found that abnormal vibration senses are the most common neurological signs in patients with cirrhosis, suggesting that peripheral nerve damage may be related to the stimulation of predominantly Aβ fibers [14, 15]. However, one study on PN in chronic liver disease using quantitative sensory testing found that cold-detection abnormalities were greater than vibration threshold abnormalities, indicating that more Aδ fibers were involved than Aβ fibers [2]. Another study on peripheral nerves in end-stage liver disease using standard nerve conduction studies and thermal detection thresholds found that 4 of 11 (36%) patients had definite abnormalities in large-fiber nerve conduction and 10 of 11 (91%) had abnormal thermal perception, indicating more involvement of C fibers than large fibers [5]. The discrepancy between the results of this current study and previous studies may be due to differences in the sensitivity of the tests used to diagnose the neuropathies, as well as the recruitment of patients at different stages of cirrhosis and with different etiologies.

In our study, we compared the incidence of SPN in patients with varying degrees of liver disease severity. There was no significant difference in the prevalence of SPN among the Child-Pugh class A, Child-Pugh class B, and Child-Pugh class C groups. Kharbanda *et al*. and Jain *et al*. found comparable results in their studies [4, 16]. We compared the CPT values of various cirrhosis severity groups and found no correlation between cirrhosis severity and sensory nerve function as measured by CPT testing. Similar findings have been reported by Kharbanda *et al*. and Jain *et al*. [4, 16]. These findings suggest that liver disease is the most important cause of neuropathy. Although diabetes and alcohol consumption independently contributed to PN, we discovered that a subset of patients with nonalcoholic cirrhosis still developed PN.

However, a study by Cocito *et al*. revealed that PN was more common in patients with severe liver disease than in those with mild liver disease [17]. Chaudhry *et al*. and Mittal *et al*. found a significant correlation between the severity of neuropathy in Child-Pugh class C compared to Child-Pugh class A and Child-Pugh class B [2, 18]. These different perspectives suggest that the pathogenesis of PN in patients with cirrhosis is still unknown; however, there may be multiple mechanisms of nerve damage, including liver failure, the specific etiology of liver disease, metabolic abnormalities, and frequent comorbidities. In one study, portal-systemic shunt and hepatocellular damage were identified as significant factors in the development of hepatic neuropathy [19]. Other studies, however, have not found an increased incidence of neuropathy in patients with cirrhosis with portocaval shunts [20]. One case report suggested that abnormalities of myelin or changes in neuronal membrane function may be responsible for slow nerve impulse conduction rather than nerve fiber loss [21]. Patients with chronic liver disease exhibited segmental demyelination and remyelination in nerve biopsies [1]. One study demonstrated a mild depolarization of the resting axonal membrane potential, but it was not identified as a cause of hepatic neuropathy [5].

Our study found no correlation between cirrhosis-related biochemical markers in the blood and the CPT-measured sensory nerve threshold. Likewise, Ng *et al*. demonstrated that there was no correlation between neurophysiological indices and blood parameters [5]. However, in the patients with alcoholic cirrhosis, CPT values at 2000 Hz were positively correlated with ALT levels, whereas CPT values at 5 Hz were negatively correlated with ALP levels. CPT values in the median nerve testing at 5 Hz were positively correlated with TBiL levels in the hepatitis B with alcoholic cirrhosis group, whereas CPT values in the deep and superficial peroneal nerves were positively correlated with AST and ALP levels. In a cross-sectional study on chronic hepatitis C patients, Mapoure *et al*. found an association between serum albumin and peripheral nerve function [15]. In addition, Hendrickse *et al*. found in another study that serum bilirubin and albumin were related to peripheral nerve function in PBC patients [11]. However, Katalin *et al*. demonstrated that serum bilirubin was associated with peripheral sensory nerve function in PBC patients, not serum albumin. In addition, in their study, elevated serum AST, ALT, and ALP levels were also associated with lower CPT values [3]. This disparity may be attributable to the different sample sizes and disease causes between our study and those in the literature. A more precise correlation between the CPT values of peripheral sensory nerves in cirrhosis and blood biochemical markers requires additional research.

This research has several limitations. First, it was conducted at a single center with a relatively small number of patients with variable liver disease etiology, necessitating additional studies with larger sample sizes. Second, these data were derived from hospitalized patients with more severe cirrhosis, whose clinical presentation may differ from the outpatient analysis. Therefore, it is possible that the clinical profiles of patients with cirrhosis in this study do not accurately represent their actual clinical profiles.

## CONCLUSION

The findings of this study revealed that the CPT value in the patients with liver cirrhosis was significantly higher than that of healthy controls and that the SPN of liver cirrhosis manifested primarily as Aβ fiber neuropathy. The correlation between blood biochemical indicators related to cirrhosis and the sensory nerve threshold observed in this study must be confirmed in a larger sample size.

## Figures and Tables

**Fig. (1):**
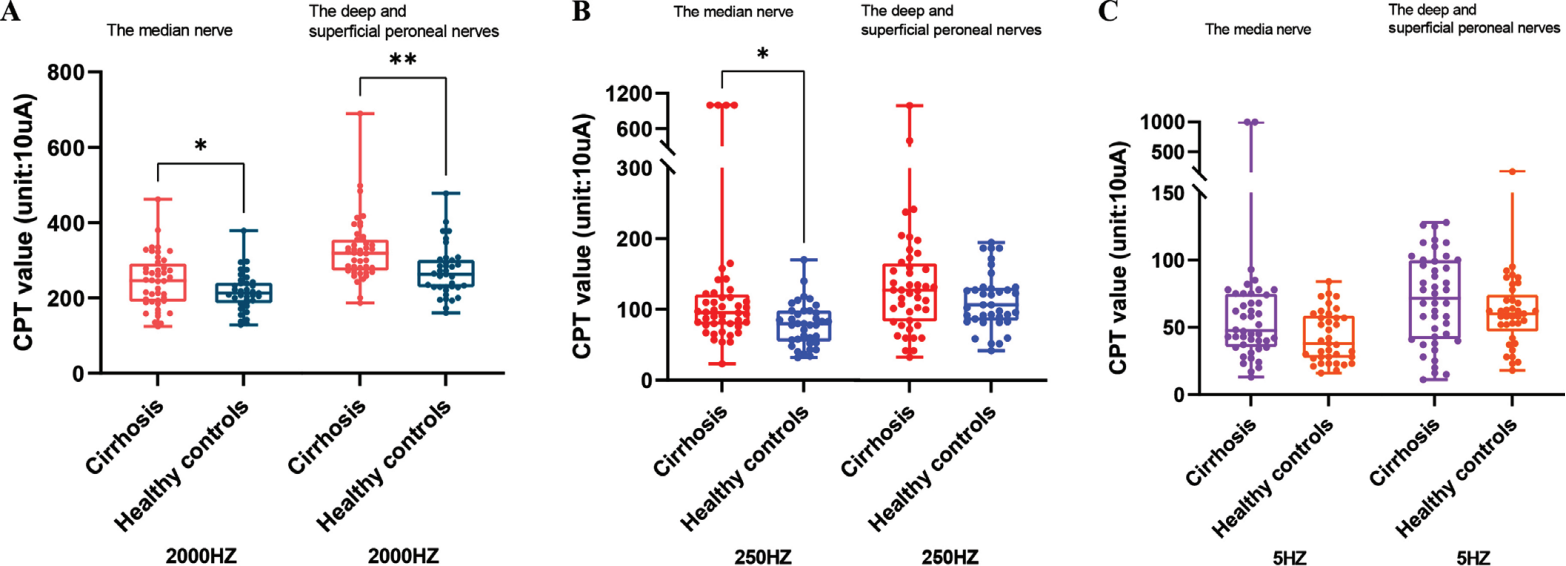
(**A**) Comparison of CPT values at 2000 Hz between the cirrhosis and healthy control groups. (**B**) Comparison of CPT values at 250 Hz between the cirrhosis and healthy control groups. (**C**) Comparison of CPT values at 5Hz between the cirrhosis and healthy control groups. H: Healthy control groups; C: study groups for cirrhosis. *p < 0.05; **p < 0.01 (not significant otherwise).

**Table 1:** Demographic data of the 44 cirrhotic patients. Data in n(%) or mean ± SD.

**Characteristic**	**n(%) / mean±SD**
Gender	
Male	33(75%)
Female	11(25%)
Age	52.52 ± 15.36
Etiology of liver cirrhosis	
Alcohol	6(13.6%)
Hepatitis B infection	24(55.5%)
More than 1(alcohol and hepatitis B)	5(11.4%)
Hepatitis C infection	1(2.3%)
Primary biliary cirrhosis	3(6.8%)
Wilsons disease	1(2.3%)
Cryptogenic	4(9.1%)
Child-Turcotte-Pugh class	
A	14(31.8%)
B	14(31.8%)
C	16(36.4%)
Complication	
Ascites	26(59.1%)
Splenomegaly	29(65.9%)
Upper gastrointestinal bleeding	15(34.1%)
Pedal edema	7(15.9%)

**Table 2:** Comparisons of the CPT values between three subgroups of cirrhosis and with healthy controls groups.

**Sensory Nerve**	**Frequency (Hz)**	**Healthy Controls (n=37)**	**Cirrhosis**		
			**Child's A (n=14)**	**Child's B (n=14)**	**Child's C (n=16)**
The median nerve	2000	215.38 ± 50.97	230.29 ± 73.65	257.36 ± 41.88	243.63 ± 91.17
	250	79.03 ± 30.59	92.50(46.00)	93.50(38.75)	101.00(59.00)
	5	38.00(31.00)	48.50 ± 18.68	53.50(39.50)	58.00(44.50)
The deep and superficial peroneal nerves	2000	263.00(72.50)	330.00 ± 53.14*	309.36 ± 73.16	319.00(84.75)*
	250	112.57 ± 40.24	154.21 ± 92.65	109.50(57.75)	131.19 ± 56.82
	5	60.00(27)	76.79 ± 36.41	69.79 ± 27.67	66.50 ± 36.34

**Note:** * *p*<0.05 *vs*. Healthy controls.

## Data Availability

The datasets used and/or analysed during the current study are available from the corresponding author upon reasonable request.

## LIST OF ABBREVIATIONS

ALP: Alkaline Phosphatase
ALT: Alanine Aminotransferase
AST: Aspartate Aminotransferase
CPT: Current Perception Threshold
CTP: Child-turcotte-pugh
PBC: Primary Biliary Cirrhosis
PN: Peripheral Neuropathy
PT: Prothrombin Time
SPN: Sensory Peripheral Neuropathy
TBil: Total Bilirubin

## STANDARD OF REPORTING

STROBE guidelines were followed.
